# Clinical characteristics and prognosis of IgA nephropathy with predominant light chain deposition

**DOI:** 10.1080/0886022X.2026.2745216

**Published:** 2026-10-25

**Authors:** Xiang Liu, Wenhui He, Xinyu Chen, Hong Liu, Anni Wang, Xuanli Tang, Xue Jiang, Jin Yu

**Affiliations:** Department of Nephrology, Hangzhou Hospital of Traditional Chinese Medicine (Hangzhou TCM Hospital Affiliated to Zhejiang Chinese Medical University), Hangzhou, China

**Keywords:** IgA nephropathy, light chain deposition, complement activation, prognostic analysis

## Abstract

To compare the clinicopathological features, treatment response, and renal outcomes of patients with IgA nephropathy (IgAN) with κ-dominant, κ–λ co-deposition, or λ-dominant light-chain deposition. This retrospective, single-centre cohort study included 105 patients with primary IgAN who underwent their first native-kidney biopsy. Twenty-one patients with κ-dominant deposition were matched to 42 patients with κ–λ co-deposition and 42 patients with λ-dominant deposition based on age, sex, 24-hour urinary protein excretion, and baseline serum creatinine. The final matching ratio was 1:2:2. The primary renal endpoint was a sustained ≥30% increase in serum creatinine from baseline. During a median follow-up of 25 months (IQR, 18–36), 21 patients reached the renal endpoint. Time to treatment response did not differ significantly among the three groups (log-rank *p* = 0.0707). However, renal progression-free survival differed significantly among the light-chain deposition groups (log-rank *p* = 0.0046). After adjustment for sex, age, 24-hour urinary protein excretion, and baseline eGFR, a higher κ deposition ratio remained associated with a lower hazard of renal progression (HR [per 1-percentage-point increase] 0.96; 95% CI: 0.94–0.99; *p* = 0.011). A higher κ light-chain deposition ratio was associated with a lower risk of renal progression in IgAN. κ predominance may be a pathological marker of a distinct and possibly less aggressive immune-complex deposition pattern.

## Introduction

IgA nephropathy (IgAN) is one of the most common primary glomerular diseases worldwide. It is characterized by the deposition of immunoglobulin A (IgA) in the glomerular mesangium and is accompanied by varying degrees of proteinuria, hematuria, and impaired renal function. Approximately 30% to 40% of patients progress to end-stage renal disease (ESRD) within 20 to 30 years after diagnosis [1]. This trend is particularly pronounced in China and poses a significant burden on the public health care system. In 1986, Lai et al. [2] discovered and reported that the mesangial deposits in patients with IgAN mainly consist of IgA-containing λ light chains. As research has progressed, the specific manifestations of κ and λ deposition in patients with IgAN have gradually attracted attention. Previous studies suggest that the dominant deposition of light chain subtypes in patients with IgAN may involve multiple mechanisms, including differences in charge characteristics [3], changes in cell migration properties [4], and imbalances in immune regulation [5]. The differences in the clinical, pathological, and prognostic aspects of different light chain-dominant types are issues of concern in clinical practice. However, the current literature review has found only four studies regarding λ light chain dominance, and λ-dominant deposition remains controversial among researchers. One study [6] proposed that the λ light chain-dominant deposition type is a more aggressive subtype of IgAN, suggesting that it is associated with more severe clinical manifestations and a poor prognosis. Other studies [7–9] have indicated that patients with IgAN dominated by λ light chains have a prognosis similar to that of patients with light chain co-deposition IgAN. At present, there are few reports regarding the clinical and prognostic differences of IgAN dominated by different types of light chains, which requires further observation and in-depth study.

Accordingly, this study aimed to compare the clinical, pathological, treatment-related, and prognostic characteristics of patients undergoing their first native-kidney biopsy-proven primary IgAN exhibiting κ-dominant, κ-λ co-deposition, or λ-dominant light-chain deposition. We hypothesized that these deposition patterns represent distinct clinicopathological phenotypes and that κ-dominant deposition, or a higher κ deposition ratio, is associated with a lower risk of early renal function deterioration than κ-λ co-deposition or λ-dominant deposition.

## Materials and methods

### Patients and study design

In this retrospective, single-centre cohort study, we identified patients with biopsy-proven primary IgA nephropathy (IgAN) who underwent their first native-kidney biopsy at Hangzhou Traditional Chinese Medicine Hospital between May 2021 and August 2024. All patients underwent their first native-kidney biopsy at cohort entry. Patients with κ-dominant deposition were identified from the entire eligible IgAN biopsy cohort. Because κ-dominant deposition was uncommon, a matched cohort was constructed to reduce baseline imbalance and improve comparability among the light-chain deposition patterns. Propensity score matching was performed using age, sex, 24-h urinary protein excretion, and baseline serum creatinine as matching covariates. Patients with κ-dominant deposition served as the index group. Each patient with κ-dominant deposition was matched to two patients with κ-λ co-deposition and two patients with λ-dominant deposition, resulting in an overall matching ratio of 1:2:2. The resulting matched cohort was used as the primary analytical population.

This was a retrospective single-centre study. The study was approved by the Ethics Committee of Hangzhou Traditional Chinese Medicine Hospital (Approval No. 2025KLL040). The requirement for written informed consent was waived because the study used only de-identified retrospective data. The study was conducted in accordance with the Declaration of Helsinki. Our exclusion criteria were: (1) renal biopsy in individuals under the age of 18 years; (2) secondary IgAN caused by systemic diseases, such as hepatitis B virus-associated glomerulonephritis, lupus nephritis, and allergic purpura nephritis; (3) immunofluorescence (IF) data of the κ and λ light chains not available; (4) concomitant malignancy; (5) plasma-cell neoplasm; (6) incomplete key clinical or pathological data required for study eligibility, propensity-score matching, or primary outcome analyses; or (7) evidence of monoclonal gammopathy or monoclonal gammopathy of renal significance, including PGNMID, based on serum and urine immunofixation electrophoresis, serum free light-chain testing, and renal pathological findings. Any individual who meets any of the above exclusion criteria was excluded from the study.

### Patient classification based on the intensity of light chain staining

Light chains, including κ (1:100, #0198, DAKO) and λ (1:100, #0199, DAKO) chains, were assayed using direct IF staining. Frozen 2-micrometer sections were cut *via* freezing microtomy and stained with fluorescein isothiocyanate (FITC)-labelled antibodies for 1 h at room temperature. Paraffin-embedded sections (3 µm thick) were prepared for staining. Antigen retrieval was performed using heat-induced epitope retrieval (HIER) with citrate buffer and enzymatic digestion with trypsin. The remaining steps were the same as those used for IF staining of frozen tissue. After being washed with buffer, the sections were mounted in glycerin and observed under an IF microscope (BX51, Olympus). The intensity grading of IF used in this study was: -, +, ++, +++, and ++++, where an IF intensity ≥ + is defined as positive, and an IF intensity ≥ ++ is defined as strong positive. According to the scoring criteria, - and ± were scored as 0 points, + was scored as 1 point, ++ was scored as 2 points, +++ was scored as 3 points, and ++++ was scored as 4 points [10]. Electron microscopy was performed in all 105 patients to assess the ultrastructural features and distribution of electron-dense deposits.

Routine direct immunofluorescence was initially performed on frozen renal biopsy sections. In all cases showing marked κ- or λ-light-chain predominance, additional immunofluorescence was performed on formalin-fixed, paraffin-embedded sections following antigen retrieval to evaluate potentially masked immunoglobulin deposits [11]. When the frozen-section and antigen-retrieved paraffin-section findings were discordant, the latter was used to adjudicate the final light-chain deposition category.

Immunofluorescence staining was independently evaluated by two experienced renal pathologists who were blinded to clinical information and outcomes. The staining intensity of IgA, κ, and λ light chains was graded semi-quantitatively on a 0–4+ scale. Light chain deposition patterns were classified according to the relative intensity difference between κ and λ staining. Interobserver agreement for κ and λ staining intensity scores was assessed using linear weighted Cohen’s kappa coefficients.

Patients were classified into three groups according to the relative immunofluorescence staining intensities of κ and λ light chains in the mesangial region: Group 1, κ-dominant deposition, defined as κ staining at least 2+ stronger than λ staining; Group 2, κ-λ co-deposition, defined as an absolute difference of <2+ between κ and λ staining intensities; and Group 3, λ-dominant deposition, defined as λ staining at least 2+ stronger than κ staining.

### Pathological parameters

The depositions of immunoglobulin G (IgG), immunoglobulin A (IgA), immunoglobulin M (IgM), complement component 3 (C3), complement component 4c (C4c), complement component 1q (C1q), and both light chains were semiquantitatively assessed by two investigators who were blinded to the clinical and pathological findings. The patient samples included in our study were classified by two pathologists according to the Oxford Pathological Classification (MEST-C score) criteria [12]. Based on the renal pathology results, the proportions of κ and λ light chains in the total light chain deposition were calculated; that is, the κ-deposition proportion (%) = κ/(κ + λ), and the λ-deposition proportion (%) = λ/(κ + λ).

### Basic clinical data collection

General information: The patients’ basic clinical information, including sex, age, systolic blood pressure (SBP), diastolic blood pressure (DBP), mean arterial pressure (MAP), and body mass index (BMI), was recorded. In addition, specific medical data, such as whether they had a history of pre-infection, hypertension, diabetes, nephrotic syndrome (NS), acute kidney injury (AKI), and gross hematuria, were collected.

Laboratory indicators: Laboratory examinations were performed within one week prior to the patient’s renal biopsy procedure. Routine blood tests, such as white blood cell count (WBC), neutrophil count (NEU), hemoglobin (HB), and platelet count (PLT), were performed; kidney function, such as Scr, blood urea nitrogen (BUN), and estimated glomerular filtration rate (eGFR) were calculated using the CKD-EPI (Chronic Kidney Disease Epidemiology Collaboration) formula; inflammation-related indicators, such as high-sensitivity C-reactive protein (hs-CRP); immune-related indicators, such as IgG, IgA, IgM, C3, and C4; and laboratory examination results, such as 24-h urine protein quantification, were also obtained. The eGFR was calculated using the 2021 CKD-EPI equation [13].

### Treatment plan

Treatment was individualized according to the KDIGO guidelines applicable at the time of treatment and each patient’s clinical and pathological characteristics [14,15]; Patients with urinary protein >0.5 g/d, regardless of the presence of hypertension, received ACEI/ARB treatment up to the maximum tolerated dose. After implementing supportive management for optimization, patients with a high risk of kidney disease progression (urine protein >1.0 g/d), underwent a 6-month course of glucocorticoid therapy after assessing the risk, and the addition of immunosuppressants, such as mycophenolate mofetil, was considered based on the clinical pathology and drug response. Patients with comorbidities such as hypertension, diabetes, hyperlipidemia, hyperuricemia, and other diseases, were administered the appropriate corresponding routine treatments.

### Treatment response definitions

 

1. During follow-up after kidney biopsy, the patients’ 24-hour urinary protein was quantified, serum creatinine (Scr) levels were monitored, and treatment response were classified into ‘treatment effective’ and ‘treatment ineffective’ using the following criteria:
  Complete remission: 24-h urinary protein quantification < 0.3 g/d and stable Scr compared with baseline (progression <30%).
  Partial remission: A reduction in urinary protein of ≥50%, 24-h urinary protein quantification < 1.0 g/d, and stable Scr compared with baseline.
  Treatment effective: Both complete remission and partial remission are classified as effective treatments. Treatment ineffective: Failure to achieve either complete or partial remission was defined as ineffective treatment.
2. Long-term outcomes: Renal progression was defined as a sustained ≥30% increase in serum creatinine from baseline, confirmed by at least two repeated measurements during follow-up. Patients who did not reach the renal progression were followed until their last available evaluable renal function assessment within the study period.

### Statistical analyses

All statistical analyses were performed using R software (version 4.0.4; R Foundation for Statistical Computing, Vienna, Austria). Data were imported and managed using the openxlsx and dplyr packages. Propensity scores were estimated by binary logistic regression using age, sex, 24-h urinary protein excretion, and baseline serum creatinine. With κ-dominant deposition as the index group, separate 1:2 nearest-neighbour matching procedures without replacement were conducted against the κ–λ co-deposition and λ-dominant groups using the MatchIt package, resulting in an overall ratio of 1:2:2.

The normality of continuous variables was assessed using the Shapiro-Wilk test, supplemented by visual inspection of Q-Q plots. Normally distributed variables are presented as means ± standard deviations (x̅ ± s) and were compared among the three groups using one-way analysis of variance. Non-normally distributed variables are presented as medians (P25, P75), and were compared using the Kruskal-Wallis test. The Benjamini-Hochberg method was used to adjust for multiple comparisons. Categorical variables are presented as number (percentage) and were compared using Pearson’s chi-square test or Fisher’s exact test, as appropriate.

Kaplan-Meier curves, log-rank tests, and Cox proportional hazards models were performed using the survival and survminer packages. RMST was estimated at both 30 and 50 months. The 30-month horizon was selected to provide an estimate within a follow-up period with a larger number of patients remaining at risk, whereas the 50-month RMST was retained as a longer-term descriptive estimate. Given the limited number of renal progression events, covariates were selected *a priori* based on clinical relevance, and sequential Cox models with increasing levels of adjustment were fitted. Results are reported as hazard ratios with 95% confidence intervals. ROC analyses were performed using pROC, with optimal cutoffs determined by the Youden index. Forest plots were generated using forestplot. All tests were two-sided, with *p* < 0.05 considered statistically significant. There were no missing data for variables included in the primary analyses. A small number of ancillary pathological variables had missing observations and were analyzed using available-case denominators without imputation.

## Results

### Comparison of general data

A total of 989 patients with primary IgA nephropathy (IgAN) were identified based on the previously described inclusion and exclusion criteria. This study used the κ-dominant deposition group as a reference. Propensity score matching was applied to construct comparable cohorts of IgAN-κ, IgAN-λ, and κ-λ co-deposition patients at a 1:2:2 ratio, adjusting for age, sex, 24-h urinary protein and serum creatinine. Ultimately, 105 patients were included in the study cohort, with 21 patients in Group 1 and 42 patients each in Group 2 and Group 3. The specific flowchart is shown in Figure 1. High-resolution immunofluorescence images and corresponding scoring examples for different light-chain deposition patterns are shown in Figure 2.

**Figure 1.**
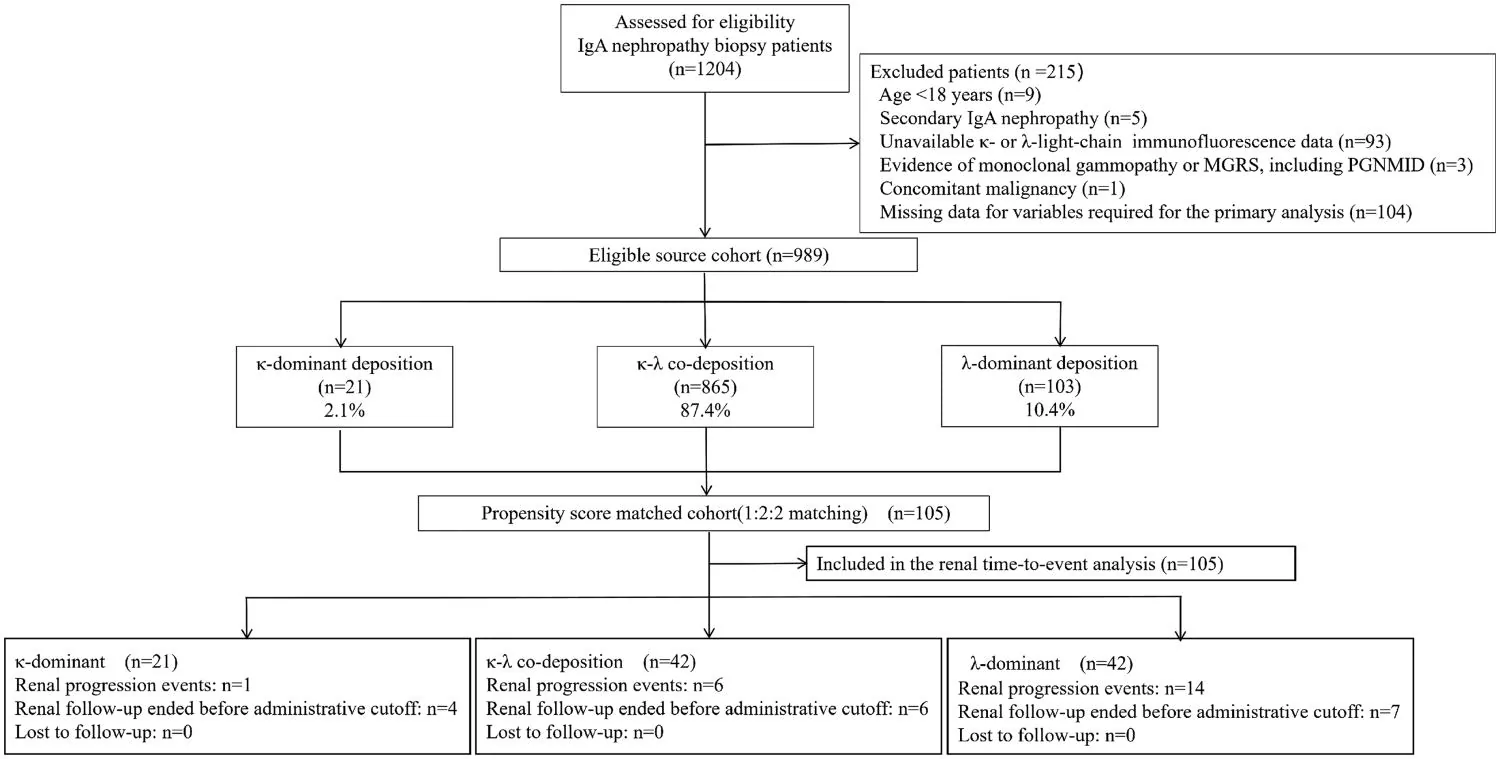
Technical roadmap. *Note:* No patients were lost to follow-up in any group. Seventeen patients without renal progression were censored at their last evaluable renal function assessment before the administrative study cutoff, including 4 in the κ-dominant group, 6 in the κ-λ co-deposition group, and 7 in the λ-dominant group.

**Figure 2.**
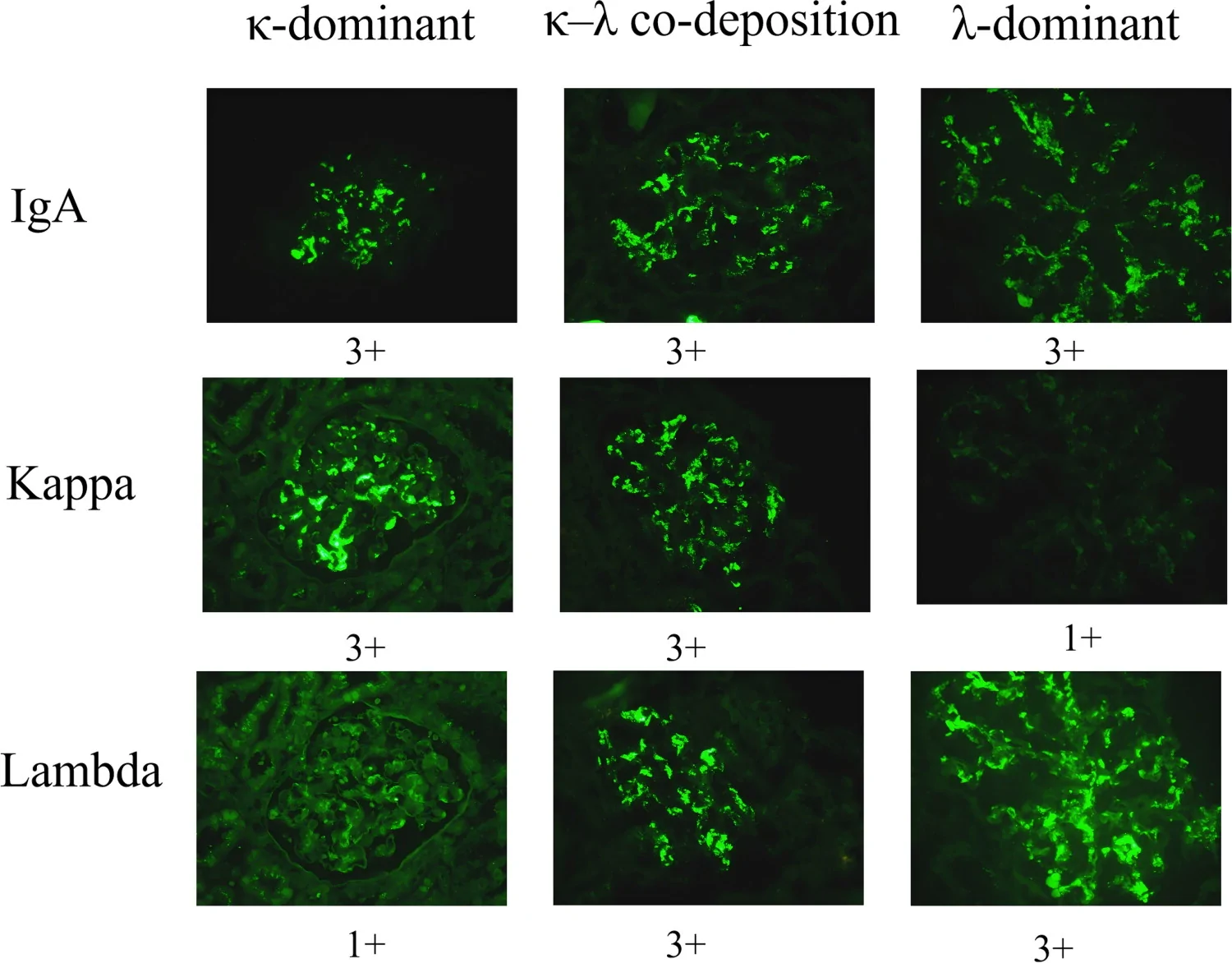
Representative high-resolution immunofluorescence images of κ-dominant, λ-dominant, and κ-λ co-deposition patterns with corresponding semiquantitative scores (Immunofluorescence, original magnification: x400).

The reproducibility of light chain intensity assessment between the two renal pathologists was evaluated using linear weighted Cohen’s kappa analysis. The agreement was substantial for κ light chain staining (weighted κ = 0.777, 95% CI 0.689–0.864; *p* < 0.001) and λ light chain staining (weighted κ = 0.845, 95% CI 0.769–0.922; *p* < 0.001).

After matching, no significant differences were observed among the three groups in terms of demographic data or the incidence of comorbidities (*p* > 0.05). Detailed information is presented in Table 1.

**Table 1.** Comparison of baseline characteristics among the three patient groups after matching.

Variable	κ-dominant(*n* = 21)	κ-λ co-deposition(*n* = 42)	λ-dominant(*n* = 42)	Overall(*n* = 105)	*P* value
Age (years)	42 ± 10	45 ± 10	44 ± 12	44 ± 11	0.653
Sex, n (%)					1.000
Male	5 (23.81)	10 (23.81)	10 (23.81)	25 (23.81)	
Female	16 (76.19)	32 (76.19)	32 (76.19)	80 (76.19)	
BMI (kg/m^2^)	22.72 (20.40,24.03)	23.44 (22.36,25.36)	22.24 (19.62,25.18)	23.05 (20.40, 25.24)	0.127
SBP (mmHg)	128 ± 14	132 ± 17	130 ± 18	130 ± 17	0.692
DBP (mmHg)	83 (77,87)	83 (76,91)	82 (73,85)	82 (76, 88)	0.722
MAP (mmHg)	98 (93,102)	99 (90,108)	98 (89,102)	99(90, 105)	0.775
History of Preceding Infection, n (%)					0.632
No	17 (80.95)	33 (78.57)	30 (71.43)	80 (76.19)	
Yes	4 (19.05)	9 (21.43)	12 (28.57)	25 (23.81)	
Hypertension, n (%)					0.304
No	12 (57.14)	20 (47.62)	27 (64.29)	59 (56.19)	
Yes	9 (42.86)	22 (52.38)	15 (35.71)	46 (43.81)	
Diabetes Mellitus, n(%)					0.839
No	18 (85.71)	38 (90.48)	38 (90.48)	94 (89.52)	
Yes	3 (14.29)	4 (9.52)	4 (9.52)	11 (10.48)	
Macroscopic Hematuria, n (%)					0.249
No	19 (90.48)	35 (83.33)	31 (73.81)	85 (80.95)	
Yes	2 (9.52)	7 (16.67)	11 (26.19)	20 (19.05)	
Nephrotic Syndrome (NS), n (%)					0.495
No	17 (80.95)	33 (78.57)	37 (88.10)	87 (82.86)	
Yes	4 (19.05)	9 (21.43)	5 (11.90)	18 (17.14)	
Acute Kidney Injury (AKI), n (%)					0.422
No	18 (85.71)	40 (95.24)	37 (88.10)	95 (90.48)	
Yes	3 (14.29)	2 (4.76)	5 (11.90)	10 (9.52)	
Follow-up duration (months)	27.0 (19.0, 39.0)	28.0 (20.0, 39.25)	24.5 (16.25, 30.5)	25.0 (18.0, 36.0)	0.136

*Note*: BMI, Body Mass Index; SBP, Systolic Blood Pressure; DBP, Diastolic Blood Pressure; MAP, Mean Arterial Pressure; Data are presented as mean ± standard deviation, median (25th - 75th percentile), or number (percentage), as appropriate. *P*-values represent the comparison between groups. Statistically significant differences are considered at *p* < 0.05.

### Comparison of laboratory indicators among the three groups

The serum complement C4 level (*p* = 0.020) and the blood κ/λ light chain ratio (*p* = 0.043) were different between the three groups. Specifically, the serum complement C4 level in Group 1 was significantly lower than that in Group 2, and the blood κ/λ light chain ratio in Group 1 was significantly greater than that in Group 3, with differences being statistically significant (*p* < 0.05). However, all of the laboratory indicators remained within the normal reference range, as detailed in Table 2.

**Table 2.** Comparison of laboratory parameters among the three patient groups

Variable	κ-dominant (*n* = 21)	κ–λ co-deposition (*n* = 42)	λ-dominant (*n* = 42)	Overall (*n* = 105)	*p* value
White blood cell count (10⁹/L)	6.14 (5.50,6.95)	6.72 (5.73,7.33)	6.06 (5.21,8.29)	6.57 (5.35,7.44)	0.456
Neutrophil count (10⁹/L)	3.82 (2.83,4.24)	3.60 (2.98,4.59)	3.33 (2.78,4.93)	3.60 (2.84,4.66)	0.962
Haemoglobin (g/L)	120.95 ± 13.32	124.02 ± 17.47	122.86 ± 15.19	122.94 ± 15.71	0.768
Platelet count (10⁹/L)	224.00 (193.00,248.00)	213.50 (193.75,247.00)	213.00 (179.50,258.25)	214.00 (188.00,252.00)	0.802
Serum creatinine (μmol/L)	86.00 (68.00,113.00)	80.50 (64.25,110.50)	83.50 (65.25,108.75)	82.00 (65.00, 111.00)	0.969
High-sensitivity C-reactive protein (mg/L)	0.70 (0.50,1.71)	0.99 (0.52,1.94)	0.88 (0.52,2.18)	0.90 (0.50, 2.06)	0.916
Albumin (g/L)	37.67 ± 4.65	35.64 ± 4.27	36.16 ± 3.92	36.24 ± 4.23	0.208
Blood urea nitrogen (mmol/L)	6.00 ± 1.54	6.12 ± 1.79	6.33 ± 2.05	6.18 ± 1.84	0.774
Uric acid (μmol/L)	358.00 (304.00,449.00)	352.00 (301.75,406.75)	365.00 (318.75,435.25)	360.00 (305.00,436.00)	0.84
Serum immunoglobulin A (mg/dL)	295.29 ± 83.97	303.59 ± 95.16	325.80 ± 106.11	310.59 ± 97.42	0.43
Serum immunoglobulin G (mg/dL)	1143.33 ± 265.82	1086.78 ± 256.97	1110.85 ± 285.32	1107.86 ± 268.37	0.736
Serum complement component 3 (mg/dL)	95.15 ± 14.79	100.74 ± 15.03	98.33 ± 15.34	98.65 ± 15.10	0.384
Serum complement component 4 (mg/dL)	22.80 (18.00,28.00)	28.00 (23.00,33.20)	25.10 (20.08,29.00)	26.00 (20.10,31.00)	0.020*
Estimated glomerular filtration rate (2021 CKD-EPI) (mL/min/1.73 m²)	79.05 ± 28.88	80.89 ± 27.90	81.03 ± 27.06	80.57 ± 27.50	0.961
24-hour urinary protein excretion (g/24 h)	1.14 (0.77,2.32)	1.14 (0.79,2.05)	1.15 (0.85,1.94)	1.14 (0.80,2.12)	0.974
Serum κ light chain (mg/dL)	280.00 (247.00,313.00)	266.00 (231.50,321.25)	279.00 (231.00,326.00)	276.00 (234.50,325.50)	0.873
Serum λ light chain (mg/dL)	160.67 ± 30.80	159.93 ± 37.30	168.19 ± 38.67	163.40 ± 36.51	0.558
Serum κ/λ light-chain ratio	1.81 (1.71,1.94)	1.68 (1.60,1.86)	1.69 (1.44,1.79)	1.70 (1.54,1.87)	0.043**
Urinary κ light chain (mg/dL)	5.02 (2.62,6.00)	2.59 (1.82,5.26)	2.77 (1.61,5.81)	2.71 (1.84,5.45)	0.387
Urinary λ light chain (mg/dL)	1.95 (1.42,3.05)	1.10 (0.70,2.39)	1.52 (0.85,2.84)	1.44 (0.79,2.79)	0.403
Urinary κ/λ light-chain ratio	2.10 ± 0.62	2.14 ± 0.55	1.88 ± 0.34	2.04 ± 0.51	0.142

**Note.** Data are presented as mean ± standard deviation or median (25th, 75th percentiles), as appropriate. *p* values compare the three groups. A *p* value <0.05 was considered statistically significant. *Significant pairwise difference between the κ-dominant and κ–λ co-deposition groups; **Between the κ-dominant and λ-dominant groups; ***Between the κ–λ co-deposition and λ-dominant groups (all *p* < 0.05). Abbreviations and symbols: CKD-EPI, Chronic Kidney Disease Epidemiology Collaboration; κ, kappa light chain; λ, lambda light chain.

### Comparison of pathological results among the three groups

The level of IgG was significantly greater in Group 3 than in Group 1 (*p* < 0.05). Although the proportion of strong C3 deposition among the groups did not reach statistical significance, a clear increasing trend was observed (76.19% → 85.71% → 95.24%, *p* = 0.086). The positive rate of C1q was similar between Group 1 (19.05%) and Group 3 (19.51%), both of which were higher than that in Group 2 (7.14%), as detailed in Table 3.

**Table 3.** Comparison of pathological features among the three patient groups.

Variable	Group 1(*n* = 21)	Group 2(*n* = 42)	Group 3(*n* = 42)	Total(*n* = 105)	*P*-value
Mesangial Hypercellularity (M), (n,%)					1.000
M0	0 (0.00)	0 (0.00)	0 (0.00)	0 (0.00)	
M1	21 (100.00)	42 (100.00)	42 (100.00)	105 (100.00)	
Endocapillary Hypercellularity (E), (n,%)					0.089
E0	15 (71.43)	24 (57.14)	18 (42.86)	57 (54.29)	
E1	6 (28.57)	18 (42.86)	24 (57.14)	48 (45.71)	
Segmental Glomerulosclerosis (S), (n,%)					0.956
S0	4 (19.05)	9 (21.43)	8 (19.05)	21 (20.00)	
S1	17 (80.95)	33 (78.57)	34 (80.95)	84 (80.00)	
Tubular Atrophy/Interstitial Fibrosis (T), (n,%)					0.261
T0	12 (57.14)	31 (73.81)	32 (76.19)	75 (71.43)	
T1/T2	9 (42.86)	11 (26.19)	10 (23.81)	30 (28.57)	
Crescent Formation (C), (n,%)					0.018***
C0	6 (28.57)	21 (50.00)	9 (21.43)	36 (33.33)	
C1/C2	15 (71.43)	21 (50.00)	33 (78.57)	69 (66.67)	
IgA Deposition Score n(%)					0.534
2	1 (4.76)	3 (7.14)	1 (2.38)	5 (4.76)	
3	19 (90.48)	39 (92.86)	40 (95.24)	98 (93.33)	
4	1 (4.76)	0 (0.00)	1 (2.38)	2 (1.91)	
IgG Deposition Score n (%)					0.027**
0	16 (76.19)	20 (47.62)	17(40.48)	53 (50.48)	
1	4 (19.05)	18 (42.86)	24(57.14)	46 (43.81)	
2	1(4.76)	4 (9.52)	1(2.38)	6 (5.71)	
IgM Deposition Score					0.363
0	1 (4.76)	1 (2.38)	3 (7.14)	5 (4.76)	
1	19 (90.48)	31 (73.81)	31 (73.81)	81 (77.14)	
2	1 (4.76)	10 (23.81)	7 (16.67)	18 (17.14)	
3	0 (0.00)	0 (0.00)	1 (2.38)	1 (0.95)	
C3–Strong Deposition, n (%)					0.086
No	5 (23.81)	6 (14.29)	2 (4.76)	13 (12.38)	
Yes	16 (76.19)	36 (85.71)	40 (95.24)	92 (87.62)	
C4–Positive, n/N (%)					1.000
No	18/19 (94.74)	38/40 (95.00)	39/41 (95.12)	95/100 (95.00)	
Yes	1/19 (5.26)	2/40 (5.00)	2/41 (4.88)	5/100 (5.00)	
C1q–Positive, n/N (%)					0.220
No	17/21 (80.95)	39/42 (92.86)	33/41 (80.49)	89/104 (85.58)	
Yes	4/21 (19.05)	3/42 (7.14)	8/41 (19.51)	15/104 (14.42)	
Percentage of Sclerotic Glomeruli (%)	17.65 (6.90,33.33)	20.00 (6.09,29.02)	13.14 (4.66,23.08)	18.18 (5.56, 29.17)	0.588
Presence of Neutrophil Infiltration, n/N (%)					0.520
No	14/20 (70.00)	23/42 (54.76)	25/42 (59.52)	62/104 (59.62)	
Yes	6/20 (30.00)	19/42 (45.24)	17/42 (40.48)	42/104 (40.38)	
Presence of Fibrous Crescents, n (%)					0.126
No	8 (38.10)	20 (47.62)	11 (26.19)	39 (37.14)	
Yes	13 (61.90)	22 (52.38)	31 (73.81)	66 (62.86)	
Presence of Cellular Crescents, n (%)					0.055
No	6 (28.57)	21 (50.00)	11 (26.19)	38 (36.19)	
Yes	15 (71.43)	21 (50.00)	31 (73.81)	67 (63.81)	

Note: Data are presented as frequency (%) for categorical variables, or as median (P25, P75) for non-normally distributed data. P values indicate comparisons among the groups. A P value less than 0.05 was considered statistically significant. *indicates a statistically significant difference between Group 1 and Group 2 (*p* < 0.05), ** indicates a statistically significant difference between Group 1 and Group 3 (*p* < 0.05), ***indicates a statistically significant difference between Group 2 and Group 3 (*p* < 0.05). For variables with missing data, values are presented as n/N (%), where N represents the number of patients with available data. Percentages were calculated using available observations as the denominator. Data were available for C4, *n* = 100 (19/40/41; 5 missing); C1q, *n* = 104 (21/42/41; 1 missing); and neutrophil infiltration, *n* = 104 (20/42/42; 1 missing). Percentages were calculated using available observations as the denominator.

In terms of MEST-C pathological classification, the C1/C2 ratio was significantly different between the groups (*p* = 0.018), with the crescent formation rate in Group 3 being significantly greater than that in Group 2 (*p* < 0.05).

The proportions of cellular crescents were 71.43% (15/21), 50.00% (21/42), and 73.81% (31/42) in Groups 1, 2, and 3, respectively. Although the proportion was numerically higher in Group 1 than in Group 2, the pairwise difference was not statistically significant after adjustment for multiple comparisons.

### Comparison of treatment protocols among the three groups

The proportions of patients receiving immunosuppressive treatment (such as corticosteroids or immunosuppressive agents) among the three groups was not significantly different (*p* > 0.05). Detailed information is provided in Table 4.

**Table 4.** Comparison of treatment protocols among the three groups.

Variable	Group 1(*n* = 21)	Group 2(*n* = 42)	Group 3(*n* = 42)	Total(*n* = 105)	*P*-value
Use of Steroids or Immunosuppressants, n(%)					0.864
No	6 (28.57)	10 (23.81)	12 (28.57)	28 (26.67)	
Yes	15 (71.43)	32 (76.19)	30 (71.43)	77 (73.33)	

### Analysis of treatment effect differences among the three groups

For the time-to-treatment-response analysis, the event was defined as the first achievement of complete or partial remission. Patients continued to be followed for subsequent renal outcomes after achieving treatment response. The Kaplan–Meier survival curve revealed that the differences in treatment effects among the three groups were not statistically significant (log-rank *p* = 0.0707). Although the overall difference was not statistically significant, the κ-dominant group showed a numerically earlier response pattern.

In contrast, Group 3 showed a delayed trend in remission. Numerical separation between the curves of the three groups was observed at 6–12 months of follow up, as shown in Figure 3.

**Figure 3.**
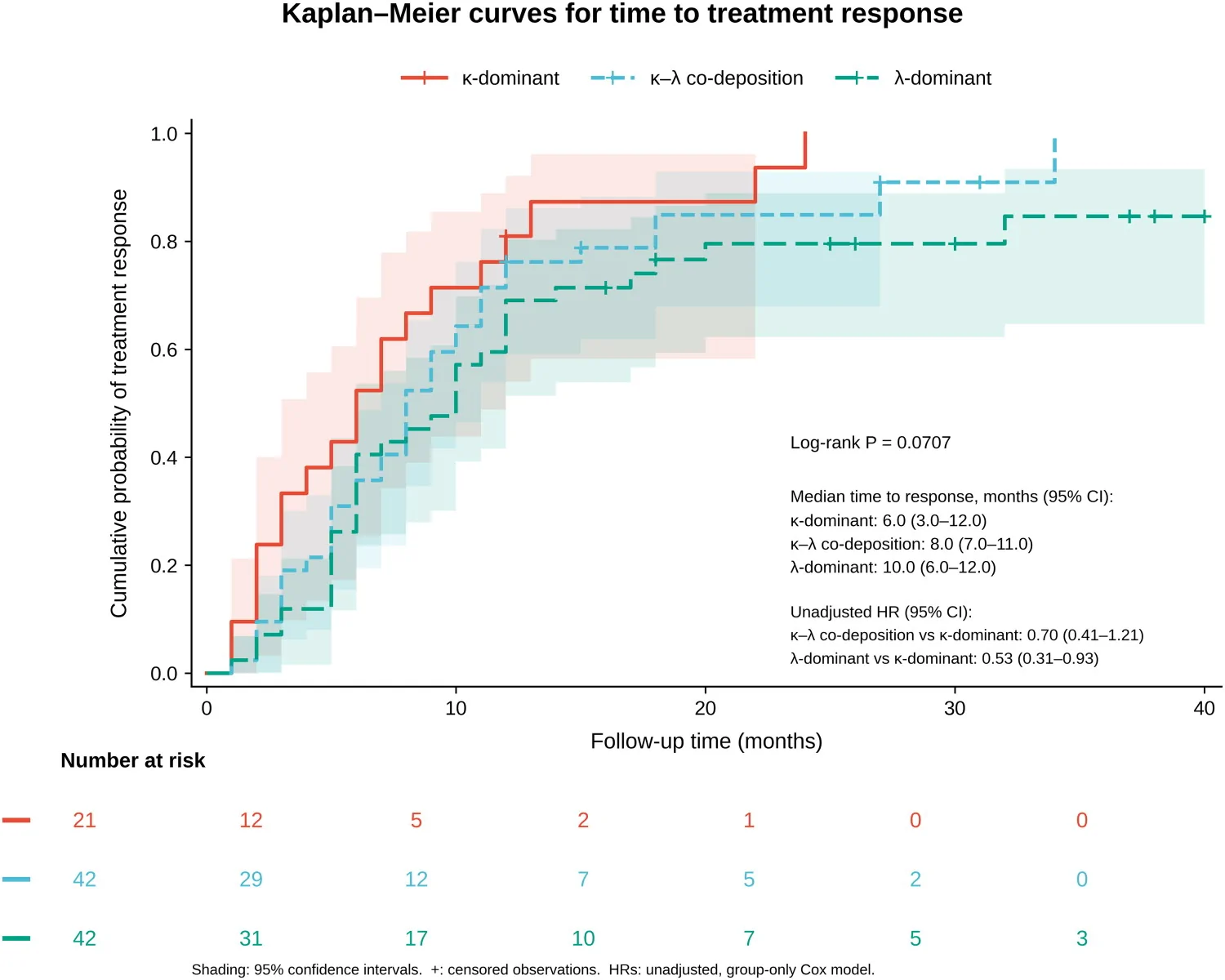
Remission survival analysis of IgAN patients with different light chain-dominant deposition patterns.

At 6 months, treatment response was achieved in 11/20 (55.0%) patients in the κ-dominant group, 16/42 (38.1%) in the κ-λ co-deposition group, and 16/42 (38.1%) in the λ-dominant group, with no significant difference among the three groups (*p* > 0.05; Figure 4A).

**Figure 4.**
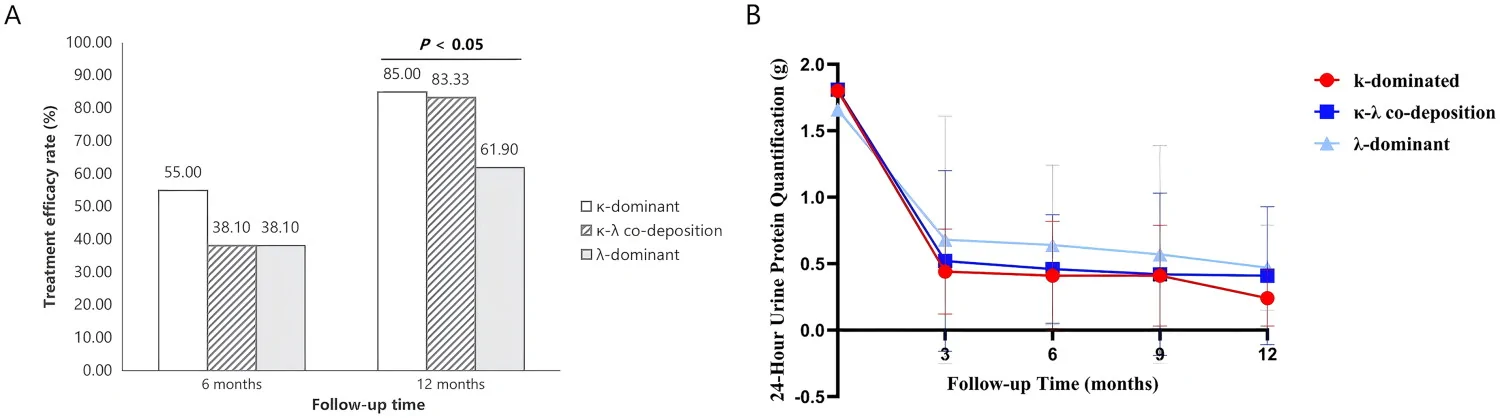
(A). Bar chart of treatment response at 6-month and 12-month follow-up among the three groups; (B). Line chart showing the dynamic changes in 24-h urine protein excretion over time in the three patient groups. *Note:* Fixed-time treatment-response rates were calculated using patients with evaluable assessments at the corresponding time point as the denominator. At 6 months, treatment response was observed in 11/20 (55.0%), 16/42 (38.1%), and 16/42 (38.1%) patients in the κ-dominant, κ-λ co-deposition, and λ-dominant groups, respectively. At 12 months, the corresponding numbers were 17/20 (85.0%), 35/42 (83.3%), and 26/42 (61.9%), respectively.

At 12 months of follow up, the treatment effects were significantly different between the three groups (*p* < 0.05). At 12 months, the corresponding response rates were 17/20 (85.0%), 35/42 (83.3%), and 26/42 (61.9%), respectively. Group 1 and Group 2 exhibited higher and similar treatment efficacy rates, 85.00% and 83.33%, as shown in Figure 4A.

The three patient groups exhibited different patterns of change in proteinuria levels at various follow-up time points. During the first 3 months. Proteinuria decreased over time in all three groups. Descriptively, the κ-dominant group showed the lowest proteinuria level at 12 months. In contrast, Group 3 demonstrated limited improvement, with the highest proteinuria level among the three groups at the 12-month mark. Group 2 showed an intermediate degree of reduction, as shown in Figure 4B.

### Analysis of differences in renal progression among the three groups

Serum creatinine levels were monitored during follow-up in all 105 patients, and renal progression was defined as a sustained ≥30% increase in Scr from baseline. The score-derived κ deposition ratio took 11 distinct values in the matched cohort: 0%, 20%, 25%, 33.33%, 40%, 42.86%, 50%, 66.67%, 75%, 80%, and 100%. During a median follow-up of 25 months (IQR, 18–36), no patients were lost to follow-up in any of the three groups (0/21 in the κ-dominant group, 0/42 in the κ-λ co-deposition group, and 0/42 in the λ-dominant group). A total of 21 patients reached the renal endpoint. Renal progression occurred in 1 of 21 patients (4.8%) in the κ-dominant group, 6 of 42 patients (14.3%) in the κ-λ co-deposition group, and 14 of 42 patients (33.3%) in the λ-dominant group. Kaplan-Meier analysis showed a significant overall difference in renal progression-free survival among the three groups (log-rank *p* = 0.0046; Figure 5A). At 30 months, 9, 20, and 11 patients remained at risk in the κ-dominant, κ-λ co-deposition, and λ-dominant groups, respectively. The corresponding 30-month RMST values were 29.7 months (95% CI, 29.1–30.0), 28.5 months (95% CI, 27.1–29.7), and 26.1 months (95% CI, 24.0–28.1), respectively. At 50 months, only 2, 7, and 2 patients remained at risk, respectively; the corresponding RMST estimates were 47.9 months (95% CI, 43.1–50.0), 45.1 months (95% CI, 41.1–48.6), and 36.6 months (95% CI, 31.2–41.8). In conclusion, κ-dominant deposition was associated with a more favorable renal progression-free survival pattern than λ-dominant deposition, as shown in Figure 5 (A and B).

**Figure 5.**
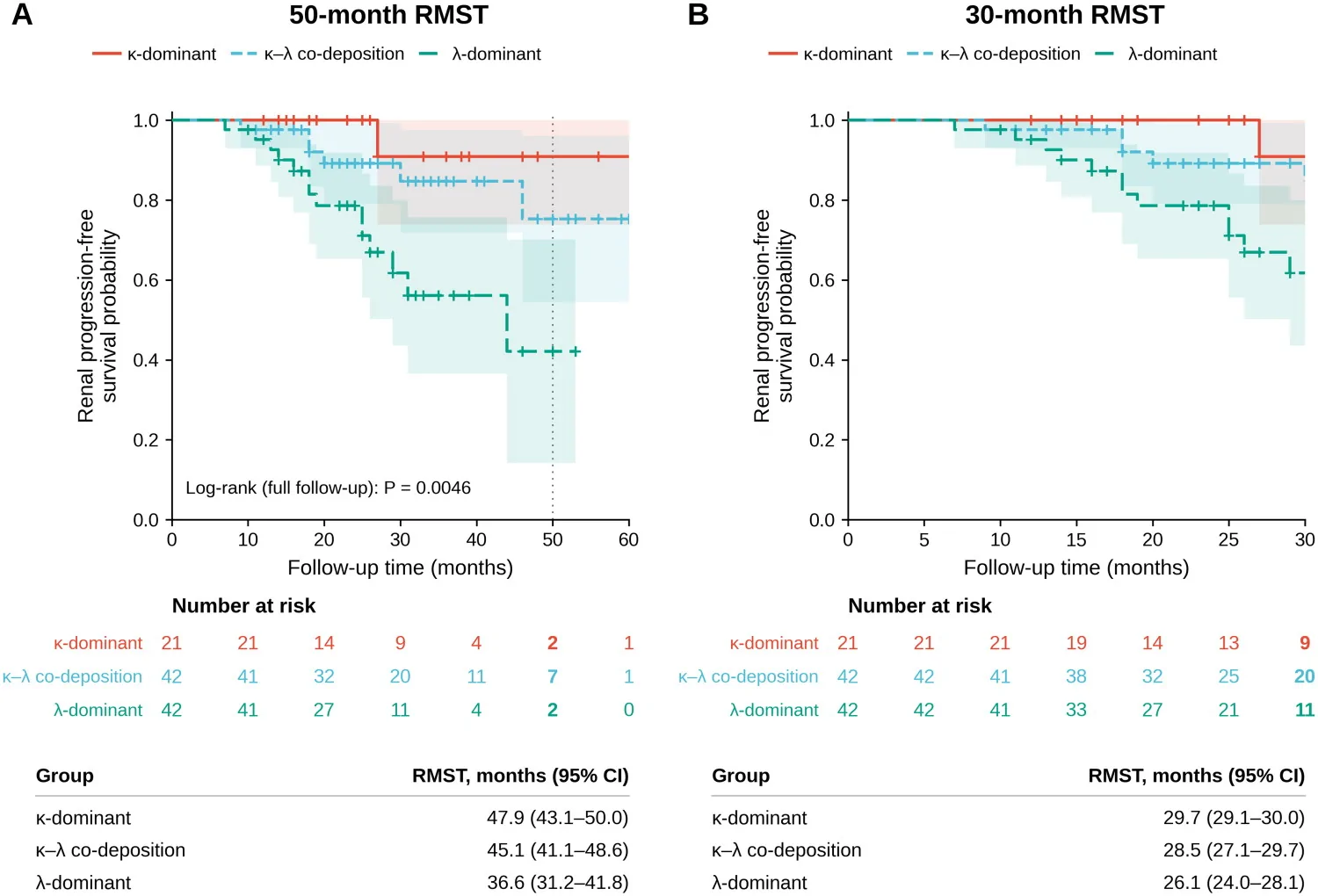
Renal progression-free survival according to light-chain deposition pattern. (A) Kaplan-Meier curves with RMST evaluated at a 50-month truncation time. (B) Kaplan-Meier curves with RMST evaluated at a 30-month truncation time. *Note:* Kaplan-Meier curves show renal progression-free survival in the κ-dominant, κ-λ co-deposition, and λ-dominant groups. Renal progression occurred in 1/21 (4.8%), 6/42 (14.3%), and 14/42 (33.3%) patients, respectively. Renal progression was defined as a sustained ≥30% increase in serum creatinine from baseline. Numbers at risk are shown below the Kaplan-Meier curves. Restricted mean survival time (RMST) estimates with 95% confidence intervals are reported at truncation times of 30 and 50 months. The P value in panel A corresponds to the overall log-rank comparison across the three groups over the full available follow-up;

A total of 21 renal progression events were included in each Cox model. In the primary adjusted analysis (Model 2), each 1-percentage-point increase in the κ deposition ratio was associated with a lower hazard of renal progression (HR, 0.96; 95% CI, 0.94–0.99; *p* = 0.011). When expressed per 10-percentage-point increase, the corresponding HR was 0.69 (95% CI, 0.51–0.92; *p* = 0.011). The association remained directionally consistent in the more extensively adjusted exploratory sensitivity analyses (Model 3: HR, 0.96; 95% CI, 0.93–0.99; *p* = 0.005; Model 4: HR, 0.96; 95% CI, 0.93–0.99; *p* = 0.005). These results are shown in Table 5 and Figure 6.

**Figure 6.**
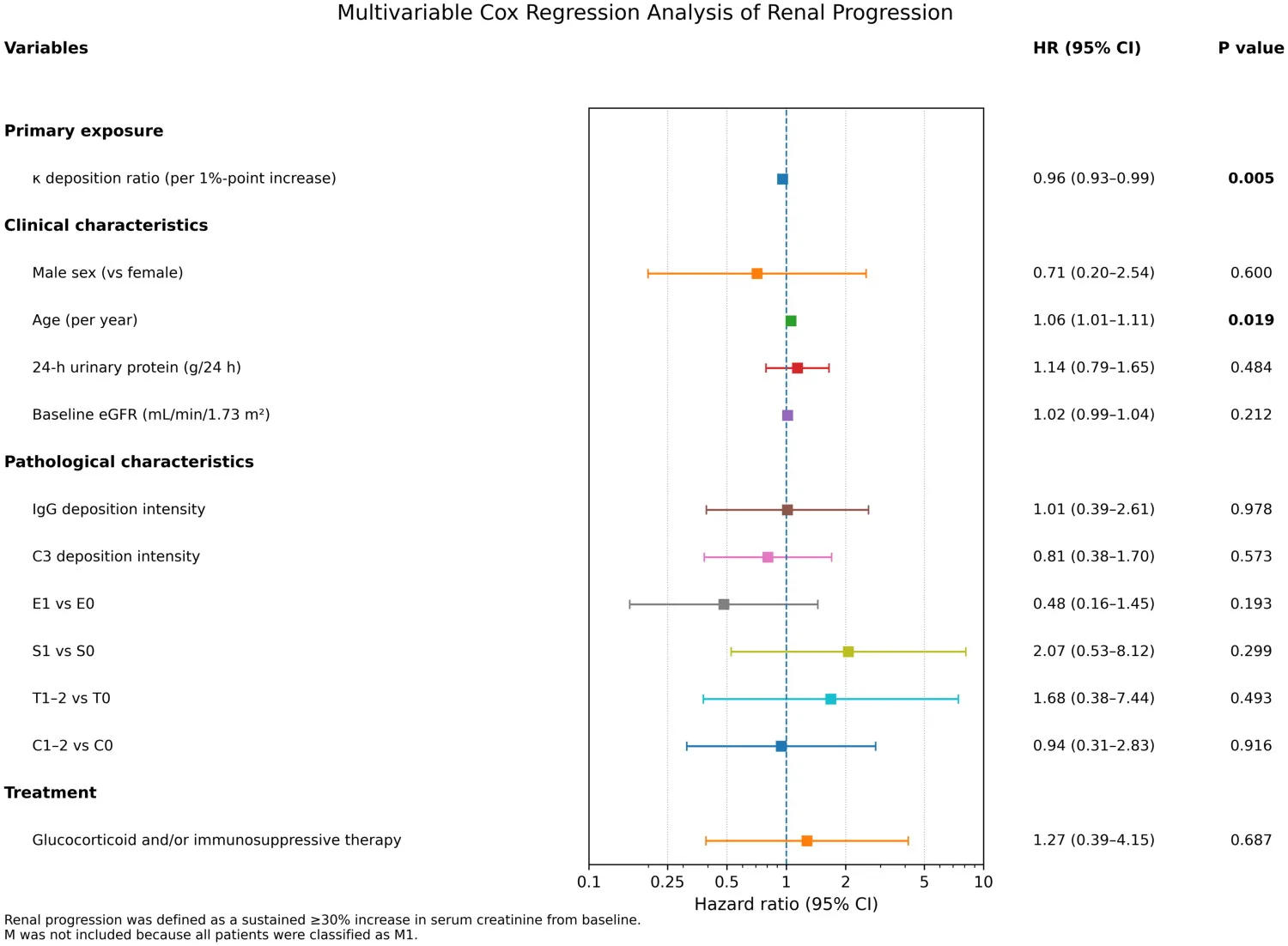
Forest plot of multivariable cox regression analysis for renal progression. *Note:* Renal progression was defined as a sustained ≥30% increase in serum creatinine from baseline.

**Table 5.** Cox Regression analysis of light chain deposition and risk of renal progression.

Variables	Model 1		Model 2		Model 3		Model 4	
	HR (95%CI)	*P*	HR (95%CI)	*P*	HR (95%CI)	*P*	HR (95%CI)	*P*
κDeposition ratio (%), per 1-percentage-point increase	0.97 (0.94–0.99)	**0.004**	0.96 (0.94–0.99)	**0.011**	0.96 (0.93–0.99)	**0.005**	0.96 (0.93–0.99)	**0.005**
κDeposition ratio (%), per 10-percentage-point increase	0.71 (0.56–0.90)	**0.004**	0.69 (0.51–0.92)	**0.011**	0.66 (0.49–0.88)	**0.005**	0.65 (0.48–0.88)	**0.005**

Abbreviations: HR, hazard ratio; CI, confidence interval.

Model definitions.

Model 1: Crude model.

Model 2 (primary adjusted model): Adjusted for sex, age, 24-h urinary protein excretion, and baseline estimated glomerular filtration rate (eGFR).

Model 3 (exploratory sensitivity analysis): Adjusted for variables in Model 2 plus IgG deposition intensity, C3 deposition intensity, and Oxford MEST-C pathological components, including E, S, T, and C scores.

Model 4 (exploratory sensitivity analysis): Adjusted for variables in Model 3 plus glucocorticoid and/or immunosuppressive therapy exposure.

All models included 105 patients and 21 renal progression events. Penalized estimation was not used. Given the limited number of renal progression events, Model 2 was designated as the primary adjusted Cox model. Models 3 and 4 incorporated additional pathological and treatment-related variables, respectively, and were considered exploratory sensitivity analyses. Penalized estimation was not used. This clarifies the adjustments made in each model for the Cox regression analysis.

*Note*: The score-derived κ deposition ratio was calculated as [κ/(κ + λ)] × 100 using the semiquantitative immunofluorescence intensity scores. Hazard ratios are presented per 1-percentage-point and per 10-percentage-point increase in the κ deposition ratio. The M component was not included because all patients were classified as M1 and therefore had no variability.

Furthermore, ROC analysis demonstrated that a lower κ deposition ratio had moderate discriminatory ability for renal progression, with an AUC of 0.707 (95% CI, 0.583–0.832; *p* = 0.003). The maximum Youden index was 0.298, corresponding to a κ deposition ratio cutoff of ≤29.17%, with a sensitivity of approximately 61.9% and a specificity of 67.9%, as illustrated in Figure 7.

**Figure 7.**
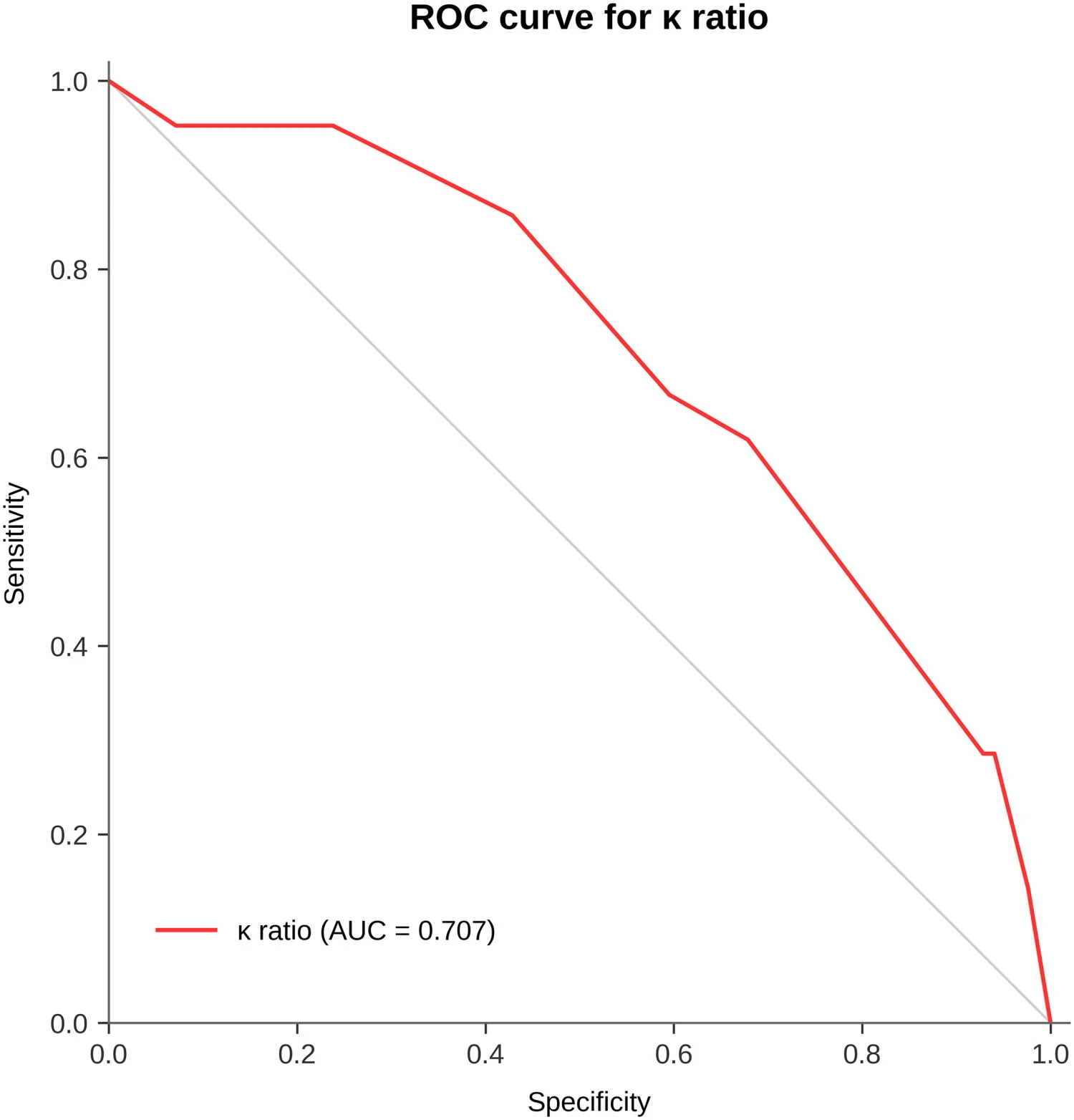
ROC curve of the κ light-chain deposition ratio for discriminating renal progression. *Note:* Renal progression was defined as a sustained ≥30% increase in serum creatinine from baseline.

## Discussion

Among 1,204 patients with biopsy-proven IgAN who were screened, 989 met the eligibility criteria, and 105 were included in the propensity-score-matched analysis. Based on mesangial light-chain deposition patterns, 21 patients (2.12%) were classified as κ-dominant, 865 (87.46%) as κ-λ co-deposition, and 103 (10.41%) as λ-dominant. Because κ-dominant deposition was uncommon, a matched cohort was subsequently constructed, including 21 κ-dominant patients, 42 κ-λ co-deposition patients, and 42 λ-dominant patients for comparative analyses. During follow-up, renal progression occurred in 1/21 (4.8%) patients in the κ-dominant group, 6/42 (14.3%) in the κ-λ co-deposition group, and 14/42 (33.3%) in the λ-dominant group.

The observation of distinct κ- or λ-dominant patterns in our cohort, after the rigorous exclusion of monoclonal gammopathy–related renal lesions, raises intriguing questions regarding the biological basis of these phenotypes. While the precise mechanisms remain to be fully elucidated, we propose that these patterns may reflect the selective expansion of specific antigen-experienced IgA-producing B-cell clonotypes or inherent differences in immunoglobulin light-chain variable-region usage. Such clonal restrictions could theoretically influence antigen specificity, immune-complex assembly, molecular size, Molecular surface charge and clearance rates, or alter mesangial binding affinity and complement activation potential.

These factors, individually or in combination, may generate light-chain deposition phenotypes with distinct clinicopathological associations.

Previous studies have indicated that the aggregation state, charge characteristics, and light chain type of IgA immune complexes may influence their deposition patterns in the glomerular mesangium [2,16]; that is, compared with κ light chain IgA, λ light chain IgA has a stronger negative charge, which may facilitate its binding to the mesangium, thereby exacerbating the immunopathological process of IgAN [17,18]. In addition, genes [5] and mucosal immunity [4,19]also play important roles in the deposition mechanism.

Furthermore, the cellular origin of these pathogenic IgA deposits warrants consideration within the context of current IgAN pathogenesis models. Dysregulated mucosal immune responses are known to promote the generation of galactose-deficient IgA1 (Gd-IgA1). Previous studies have shown that [20], plasma cells from the tonsils and gastrointestinal tract primarily secrete λ-light chain IgA, whereas plasma cells from the bone marrow predominantly produce κ-light chain IgA.

Mucosally activated B cells or plasmablasts may migrate to systemic sites, including the bone marrow, where they may become long-lived plasma cells and contribute to sustained production of circulating polymeric IgA1. The mucosal and bone marrow sources of pathogenic IgA may represent interconnected stages rather than separate pathways. Mucosal immune activation may occur before the migration and persistence of IgA-producing cells in systemic tissues or the bone marrow, which may contribute to different IgA deposition patterns.

A recent study [4] further revealed that, compared with κ-light chain-positive Gd-IgA1 B cells, λ-light chain-positive Gd-IgA1 B cells are more likely to highly express CCR10 and CCR9. These binding receptors facilitate the migration of B cells to the mucosal tissues of the upper respiratory and gastrointestinal tracts, providing additional evidence that the development of IgAN may be associated with immune stimulation in these areas. In addition, a study by Su et al. [5] revealed that in intestinal IgA plasma cells, λ-light chains undergo more frequent gene rearrangement and modifications, whereas κ-light chains, due to limitations in their genomic structure, rarely experience similar modifications. This suggests that κ-light chains are more stable during the antibody generation process. This stability may help maintain antibody specificity, enhance the immune system’s adaptability to the gut microbiome, and reduce excessive immune responses.

However, it is important to interpret these possibilities with caution. The hallmark abnormal O-glycosylation of Gd-IgA1 occurs within the hinge region of the IgA1 heavy chain, and direct evidence demonstrating that Gd-IgA1-producing B-cell subsets preferentially express specific κ or λ light chains is currently lacking. Moreover, as our study did not include paired heavy- and light-chain repertoire sequencing or tissue-specific B-cell profiling, we cannot definitively determine whether the observed deposition patterns originated predominantly from mucosal or bone marrow plasma-cell compartments.

Consequently, we frame the association of  κ predominance not as evidence of a direct protective effect, but as a potential marker of a distinct, possibly less aggressive immune-complex phenotype. Future investigations integrating single-cell paired heavy- and light-chain B-cell receptor sequencing across mucosal tissues, peripheral blood, and bone marrow-coupled with Gd-IgA1-specific characterization and proteomic analysis of glomerular deposits-will be essential to clarify the cellular and molecular origins of these light-chain dominance phenotypes.

This study revealed that different light chain dominant types of IgA nephropathy (IgAN) may involve distinct complement activation patterns. The activation of the complement system plays a crucial role in IgA nephropathy (IgAN), primarily through the classical pathway (CP), alternative pathway (AP), and lectin pathway (LP). In this study, the κ-dominant deposition group presented significantly less IgG deposition than the λ-dominant deposition group (*p* < 0.05). The results of this study revealed a slight difference in the proportion of strong C3 deposition between the κ and λ groups (76.19% vs. 95.24%, *p* = 0.086). The limited sample size and relatively short follow-up period may have reduced the statistical power of the analysis and impacted the robustness of the conclusions. Moreover, both the κ-dominant group and the λ-dominant group presented C1q deposition rates of 19.05% and 19.51%, respectively, which were higher than those of the κ-λ co-deposition group (7.14%). Previous studies have shown that IgG can activate the classical complement pathway by binding to C1q, enhancing the formation of C3 convertase and thereby increasing complement activation [21,22]. This may explain why, in the λ group, with greater IgG deposition, the classical complement pathway was more strongly activated, leading to more significant C3 deposition. In contrast, the κ group has less IgG deposition, and the activation of the classical complement pathway may be weaker than that in the λ group, leading to relatively lower C3 deposition. The difference in the deposition intensity between C1q and C3 suggests that the classical pathway (CP) alone may not fully explain the significant deposition of C3, and the alternative pathway (AP) likely plays a dominant role in this process.

From a clinical perspective, λ-dominant deposition with greater IgG co-deposition may represent a distinct immune-complex phenotype potentially associated with increased IgG-containing immune complexes and complement activation. These patients may be a potential subgroup for future clinical trials of complement-targeted therapies. However, glomerular IgG deposition alone cannot be used as a validated marker to predict response to complement inhibitors or to determine the specific complement pathway involved. Future studies combining light-chain deposition patterns with tissue and circulating complement biomarkers are needed to clarify whether these patients may benefit more from complement-targeted treatment.

In terms of clinical outcomes, patients with κ-dominant deposition showed a numerically more favorable treatment-response pattern, particularly between 6 and 12 months, although the overall difference in time to treatment response did not reach statistical significance. The κ-dominant group also showed the highest renal progression-free survival, whereas the λ-dominant group showed the lowest.

Interestingly, the serum κ/λ ratio differed among the three groups (*p* = 0.043), despite the exclusion of monoclonal gammopathy. This modest difference should not be interpreted as evidence of systemic monoclonality, because serum light-chain concentrations and their ratio may be influenced by renal clearance and polyclonal immunoglobulin production. The clinical significance of this difference remains uncertain and requires further investigation.

Many studies have shown that the deposition of IgG in the glomerular mesangial area is associated with a poor prognosis. Shin et al. [23] reported that glomerular IgG deposition in patients with IgAN is closely associated with renal function deterioration. Specifically, the greater the amount of IgG deposition is, the lower the patient’s cumulative renal survival rate. Moderate to severe IgG deposition can independently predict poor prognosis. A recent study by Xing et al. [24], which included a larger sample size, revealed that the incidence of composite renal events was greater in the high-IgG-deposition group than in the low-IgG-deposition group, further validating the conclusion of the current study. In addition, another study [21] revealed that, in an IgA-mediated acute glomerulonephritis model in rats, the injection of IgG alone led to transient proteinuria without significant glomerular inflammation. Injection of IgA alone induced hematuria and mild complement deposition. However, when IgA and IgG co-deposited, there was a significant increase in C5b-9 deposition, accompanied by more severe proteinuria and hematuria. These findings suggest that co-deposition of IgA and IgG can induce selective, complement-dependent glomerular inflammation, thereby exacerbating kidney damage. In addition, studies have shown that when IgG and IgA co-deposit, they are more likely to induce hematuria [25,26]. This is consistent with the trend observed in our study, the proportion of macroscopic hematuria was numerically higher in the λ-dominant group (26.19%) than in the κ-dominant group (9.52%), although the difference was not statistically significant (*p* = 0.249), it still supports the role of IgG deposition in IgAN. Bellur et al. [27] reported that patients with glomerular IgG deposition tended to have poorer kidney survival rates, although the difference did not reach statistical significance (*p* = 0.06). However, as a greater proportion of patients with positive IgG deposition received immunosuppressive treatment, this introduced a certain bias in the clinical outcome analysis. In this cohort, the κ-dominant group showed more favorable renal outcomes than the λ-dominant deposition group, which may be related to the differences in IgG deposition between the two groups at the pathological level. In conclusion, the degree of IgG-mediated complement activation in different subtypes of IgAN may vary depending on the dominance of κ and λ light chains. In the κ group, complement activation may be relatively mild, whereas in the λ group, there is a greater tendency for complement cascade amplification, leading to more severe glomerular damage. This difference could influence the clinical presentation and prognosis of patients with IgAN. Future studies should further investigate the pathogenic mechanisms of IgG deposition and explore whether interventions targeting IgG-mediated complement activation could serve as a potential therapeutic approach for IgAN.

In addition to the differences in the quantitative deposition of IgG, this study also revealed significant differences in the incidence of crescent formation (C1/C2) across the three groups in terms of the MEST-C score (*p* = 0.018). The proportions of C1/C2 lesions were 71.43% (15/21) in the κ-dominant group, 50.00% (21/42) in the κ-λ co-deposition group, and 78.57% (33/42) in the λ-dominant group. The λ-dominant group had a significantly higher proportion of C1/C2 lesions than the κ-λ co-deposition group after adjustment for multiple comparisons. Despite the similar proportions of crescents (C1/C2) in both the κ and λ groups, their prognoses were strikingly different. Specifically, the κ-dominant group showed a more favorable pattern of renal progression, whereas the λ-dominant group showed less favorable clinical outcomes. This result contradicts the traditional view that IgAN with crescentic deposition is associated with a poor prognosis [28–30]. However, an increasing number of studies have proposed a different perspective, suggesting that crescent formation may not be directly associated with the prognosis of patients with IgAN [31,32]. The findings of this study also suggest that crescent formation itself may not be a decisive prognostic factor in this cohort. Overall, the conclusions of this study regarding pathological features are similar to those of Ravipati’s team[8], which included a large, multicenter, multiethnic cohort. Both this study and Ravipati’s study agree that λ-dominant deposition is associated with increased pathological activity. However, Ravipati’s team did not report a significant difference in clinical outcomes. The researchers in that study suggested that the discrepancy between pathology and outcomes could be attributed to differences in the patient racial background, research methodology, and follow-up duration, indicating certain limitations.

Previous studies have explored the potential causes of different light chain depositions in IgAN, along with their associated clinicopathological features and possible pathogenic implications. However, research regarding the clinical outcomes and prognoses of patients with IgAN with different types of light chain deposition remains very limited. Whether differences in clinical prognosis exist among patients with varying light chain deposition patterns still requires further investigation and validation.

A higher κ deposition ratio remained significantly associated with a lower risk of renal disease progression when a sustained ≥30% increase in serum creatinine was used as the renal endpoint. Because both the score-derived κ deposition ratio and the three-group light-chain deposition classification were derived from the same κ and λ immunofluorescence intensity scores, their concordant associations should be interpreted as internally consistent findings rather than as independent lines of evidence. This finding supports the observed association when renal progression was defined using the study-specified serum creatinine-based endpoint. Nevertheless, a sustained ≥30% increase in serum creatinine remains a surrogate marker of renal deterioration rather than a definitive kidney failure endpoint, and the limited number of more advanced events, including doubling of serum creatinine and kidney failure requiring kidney replacement therapy, precluded robust analyses of irreversible kidney failure outcomes.

Our findings suggest that a higher κ deposition ratio may represent a marker of a less aggressive immune-complex deposition phenotype, as it was associated with a lower risk of renal deterioration in Cox regression analysis.

To evaluate the discriminatory ability of the κ light chain deposition ratio for renal progression, ROC curve analysis was performed.

The area under the curve (AUC) was 0.707 (95% CI, 0.583–0.832, *p* = 0.003), indicating moderate discriminative ability. The optimal cutoff value, determined by the maximum Youden’s index (0.298), corresponding to a κ deposition ratio cutoff of ≤29.17%. These results suggest that the κ deposition ratio may serve as a potential prognostic marker for risk stratification in IgAN. This study not only reinforces the significant clinical and pathological features associated with different light chain deposition patterns in IgA nephropathy, but it also offers a novel perspective for more in-depth assessment of the clinical prognosis of patients with IgAN.

## Conclusion

Patients with κ-dominant deposition showed a numerically more favorable treatment-response pattern than those with λ-dominant deposition, with numerically higher remission rates between 6 and 12 months, although the overall difference in time to treatment response was not statistically significant.

The κ-dominant group showed the highest renal progression-free survival, whereas the λ-dominant group showed the lowest.

A higher κ light-chain deposition ratio remained associated with a lower risk of renal progression after multivariable adjustment, suggesting that κ predominance may represent a pathological marker of a distinct and potentially less aggressive immune-complex deposition phenotype. However, its prognostic value requires validation in larger, multicenter cohorts.

## Limitations

Several limitations should be acknowledged. First, this was a single-centre retrospective cohort study, which may limit the generalizability of our findings to other populations and clinical settings. Second, although a 1:2:2 matching strategy was used to improve baseline comparability, the number of patients with κ-dominant deposition was small (*n* = 21), reflecting the rarity of this pathological phenotype. The number of renal progression events was also limited, particularly in the κ-dominant group, because of the small sample size and relatively stable renal function among the included patients. In addition, although electron microscopy was performed in all patients, IgG subclass staining was not available in this retrospective cohort. Therefore, the subclass composition of glomerular IgG deposits could not be determined, which limits further mechanistic interpretation of the observed IgG co-deposition patterns.

In this study, a sustained ≥30% increase in serum creatinine was used as the renal endpoint to assess renal function deterioration. Although this endpoint allowed the detection of clinically relevant renal deterioration during follow-up, it is less definitive than established hard renal outcomes such as doubling of serum creatinine, kidney failure, or the need for kidney replacement therapy. Moreover, only 21 renal progression events occurred, which limited the number of covariates that could be reliably accommodated in the Cox models. Therefore, our findings should be considered exploratory and require further validation in larger multicenter cohorts with longer follow-up and established renal endpoints.

The matched cohort also showed a higher proportion of female patients. This was mainly because the κ-dominant reference group contained more female patients and sex was included as a matching variable. Although sex matching improved the comparability among groups, it reduced the epidemiological representativeness of the matched cohort and may limit the applicability of our findings, particularly in male patients with IgAN. In addition, residual confounding cannot be completely excluded despite the use of matching. Future multicenter studies with larger sample sizes and a more balanced sex distribution are needed to confirm these findings and determine whether the observed associations differ between men and women.

Because only a few patients remained at risk during later follow-up, 30-month RMST was additionally reported to provide a more stable summary within a better-supported observation period.

## Data Availability

The data and materials supporting the results or analyses of this study are available from the corresponding author upon reasonable request.
